# A Brief Online and Offline (Paper-and-Pencil) Screening Tool for Generalized Anxiety Disorder: The Final Phase in the Development and Validation of the Mental Health Screening Tool for Anxiety Disorders (MHS: A)

**DOI:** 10.3389/fpsyg.2021.639366

**Published:** 2021-02-22

**Authors:** Shin-Hyang Kim, Kiho Park, Seowon Yoon, Younyoung Choi, Seung-Hwan Lee, Kee-Hong Choi

**Affiliations:** ^1^School of Psychology, Korea University, Seoul, South Korea; ^2^KU Mind Health Institute, Korea University, Seoul, South Korea; ^3^Department of Adolescent Psychology, Hanyang Cyber University, Seoul, South Korea; ^4^Department of Psychiatry, Inje University Ilsanpaik Hospital, Goyang, South Korea

**Keywords:** screening tests, generalized anxiety disorder, psychometrics, item response theory, diagnostic utility, online assessment

## Abstract

Generalized anxiety disorder (GAD) can cause significant socioeconomic burden and daily life dysfunction; hence, therapeutic intervention through early detection is important. This study was the final stage of a 3-year anxiety screening tool development project that evaluated the psychometric properties and diagnostic screening utility of the Mental Health Screening Tool for Anxiety Disorders (MHS: A), which measures GAD. A total of 527 Koreans completed online and offline (i.e., paper-and pencil) versions of the MHS: A, Beck Anxiety Inventory (BAI), Generalized Anxiety Disorder-7 (GAD-7), and Penn State Worry Questionnaire (PSWQ). The participants had an average age of 38.6 years and included 340 (64.5%) females. Participants were also administered the Mini-International Neuropsychiatric Interview (MINI). Internal consistency, convergent/criterion validity, item characteristics, and test information were assessed based on the item response theory (IRT), and a factor analysis and cut-off score analyses were conducted. The MHS: A had good internal consistency and good convergent validity with other anxiety scales. The two versions (online/offline) of the MHS: A were nearly identical (*r* = 0.908). It had a one-factor structure and showed better diagnostic accuracy (online/offline: sensitivity = 0.98/0.90, specificity = 0.80/0.83) for GAD detection than the GAD-7 and BAI. The IRT analysis indicated that the MHS: A was most informative as a screening tool for GAD. The MHS: A can serve as a clinically useful screening tool for GAD in Korea. Furthermore, it can be administered both online and offline and can be flexibly used as a brief mental health screener, especially with the current rise in telehealth.

## Introduction

The Lancet Global Mental Health series of articles and the World Health Organization (WHO) Mental Health Gap Action Program (mhGAP) have highlighted the importance of preventive interventions in mental health (Lancet Global Mental Health Group, 2007; World Health Organization, 2008), emphasizing the need for early screening of mental disorders and the transference of patients to psychiatric professionals (Katon and Roy-Byrne, 2007; World Health Organization, 2008). Screening tools for anxiety disorders have received less clinical attention even though the prevalence of anxiety disorders is as high as that of depression, and the use of screening tools has been relatively limited (Stein et al., 2004; Kroenke et al., 2007; Fernández et al., 2012).

Generalized anxiety disorder (GAD) is commonly observed, with a prevalence of 1.6–7.3% in primary care and of 13% in psychiatric settings (Kessler et al., 2001; Lieb et al., 2005). According to the recent World Mental Health Survey, GAD as assessed using the Diagnostic and Statistical Manual of Mental Disorders (DSM)-5 is more prevalent than GAD as assessed using the DSM-IV (the lifetime prevalence of the former is 37% higher and its 12-month prevalence is 50% higher) and GAD plays a substantial role in functional impairment (Ruscio et al., 2017). Considering the socioeconomic burden of functional impairment, low productivity, and healthcare costs associated with undiagnosed GAD (DuPont et al., 1996; Ruscio et al., 2017), the use of reliable and valid screening tools has become a high priority for efficient, economical, and early interventions.

Several screening tools designed to diagnose anxiety disorders have already been developed. The Generalized Anxiety Disorder Questionnaire-IV (GAD-Q-IV; Newman et al., 2002) and Generalized Anxiety Disorder 7-item scale (GAD-7; Spitzer et al., 2006) have performed well in identifying GAD in primary care, with good sensitivity and specificity. However, the GAD-Q-IV may be inaccurate in its severity rating due to its intrinsic flexible response format. Several psychometric weaknesses of the GAD-7 have also been reported. The GAD-7 has been reported to have poor specificity in a psychiatric setting, despite being a good screening tool in primary care (Kertz et al., 2013; Beard and Björgvinsson, 2014), and it has repeatedly demonstrated a high false positive rate (Kertz et al., 2013; Beard and Björgvinsson, 2014; Ahn et al., 2019). Due to this, it is recommended that additional clinical interviews be performed or other screening tools be administered to diagnose anxiety disorders, rather than using the GAD-7 alone (Jordan et al., 2017; Ahn et al., 2019).

Since it has been suggested that the quality of life should also be measured in assessing GAD, the Overall Anxiety Severity and Impairment Scale (OASIS) included behavioral avoidance and social impairment, factors that had been overlooked in previous tools (Campbell-Sills et al., 2009). However, the OASIS has been reported to be a good measure of impairment caused by anxiety, rather than a reliable screening tool for GAD (Ito et al., 2015). Diagnostic screening tools should reflect not only clinical symptoms but also actual functional impairments.

Screening tools should have high sensitivity and specificity while being concise, and some tests based on the item response theory (IRT) model have been recently developed. For example, computerized adaptive tests (CAT) include targeted items, have different numbers of items, and reflect individual characteristics in the scoring method—these tests utilize the advanced psychometric algorithm provided by the IRT (Gibbons et al., 2014). Most IRT-based tests developed to date have been targeted at Western individuals. As the Korean mental health policy paradigm emphasizes prevention and community-based services for early intervention, the need for a short, clinically useful screening tool for anxiety that reflects Korean characteristics has emerged. For this purpose, the researchers of this study developed an IRT-based Mental Health Screening Tool for Anxiety Disorders (MHS: A) that reflects the item response characteristics of Koreans, which could serve as a foundation for constructing a CAT-based test in the future.

Shame and stigma have been reported to be the biggest barriers to seeking mental health treatment, even higher than financial barriers (Goetter et al., 2020). In Korea, as in other Asian cultures, the stigma surrounding psychiatric services is more prominent (Chung and Kwon, 2006; Cho et al., 2009). Given that GAD patients frequently overuse non-mental health medical services (Roy-Byrne and Wagner, 2004), the MHS: A could be a useful screening tool to detect GAD when they visit primary care clinics for anxiety-related issues.

This study aimed to evaluate the psychometric properties (i.e., reliability and validity) of an IRT-based anxiety screening tool and to measure its specificity, sensitivity, and cut-off score for diagnosis. Furthermore, we compared the MHS: A with existing screening tests for anxiety (i.e., the GAD-7, Beck Anxiety Inventory [BAI, Beck et al., 1988], and Penn State Worry Questionnaire [PSWQ, Meyer et al., 1990]) regarding their ability to identify anxiety disorders, especially GAD.

## Method

### Development Procedure

The MHS: A was developed through a three-stage process over 3 years (2016–2018), and this study covers Stage 3. All stages of the scale development and validation process received ethical approval from the institutional review boards of Korea University and Ilsan Paik Hospital [1040548-KU-IRB-15-92-A-1(R-A-1)(R-A-2)(R-A-2), ISPAIK 2015-05-221-009]. The MHS: A development procedure was as follows. The details of Stages 1 and 2 of the process are covered in Kim et al. (2018).

Stage 1: Item pool generation

In the first stage, a literature review was performed and focus group interviews with GAD patients were conducted; a total of 412 preliminary item pools were constructed. We classified each item into nine areas of the GAD diagnostic criteria (including problems with functioning as one separate area) and three levels of symptom difficulty. A preliminary validation was conducted with 153 healthy individuals and 101 individuals with GAD.

Stage 2. Items selection

Based on the results of the previous preliminary test, 172 items were included in the final item pool in the second stage. A total of 613 participants took the MHS: A and other anxiety tests and were interviewed using the Mini-International Neuropsychiatric Interview (MINI). To avoid bias, an interviewer conducted the MINI while blinded to the participants' diagnostic information and anxiety assessment scores and vice versa. After examining for validity, we selected the best combination of items to screen GAD, and 11 items were chosen as the final MHS: A items.

Stage 3. Final validation and online version development

**Figure 1:**
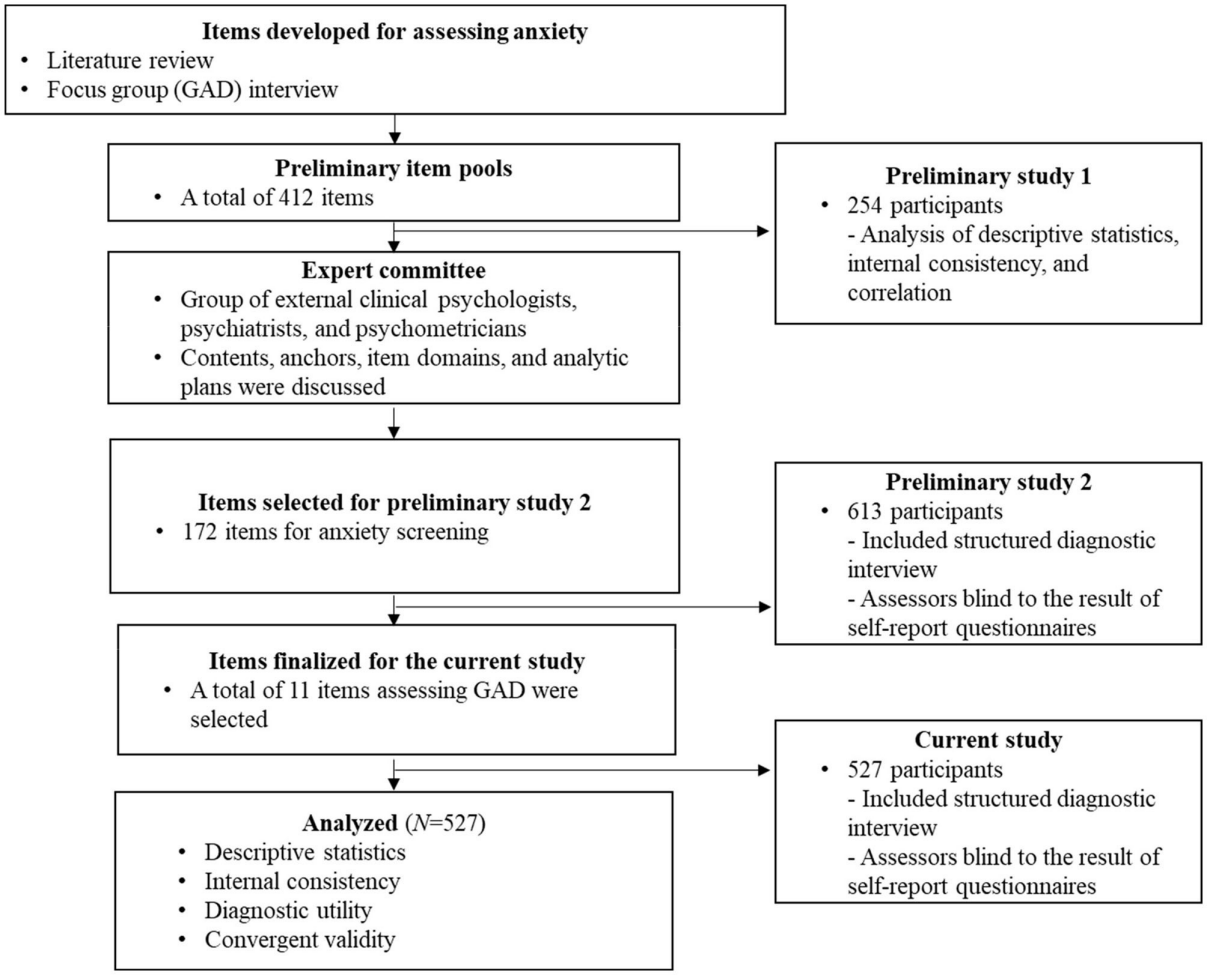
The development procedure.

To validate the final version of the MHS: A, data were collected in the same manner as in the second-year study. A total of 544 individuals were recruited for the study through online recruitment advertisements and visits to university hospitals in Seoul and Goyang. The assessment included the MINI, MHS: A, GAD-7, PSWQ, and BAI. The MHS: A implemented online scoring due to the weight of each item, and an online platform was developed to enable assessment and scoring. In this study, both the paper-and-pencil and online versions of the MHS: A were utilized, and each of the two versions was placed at the beginning and end of the entire test so that results would be less affected by the repetition effect. Both versions were administered to all participants, and the scales were presented in the same order. A total of 527 people completed the tests, including both versions of the MHS: A. The weights between items were used to calculate the average of the difficulty values, based on the polytomous IRT analysis. See Figure 1 for a visual representation of the phases.

### Participants

A total of 527 individuals participated in the current study between 2017 and 2018. Among these participants, 270 were recruited from college hospital visitors using consecutive sampling. The rest were randomly recruited via an online advertisement. The participants from the hospitals included both clinical (e.g., psychiatric or non-psychiatric patients) and healthy samples. Similarly, the participants recruited via the online advertisement included both clinical and healthy samples. Our exclusion criteria included participants who: (1) provided inappropriate responses, (2) had a history of surgery, (3) had other severe disorders, or (4) were below 19 years of age. All the participants included in the current study participated voluntarily and signed written informed consent forms. The remuneration provided to the study participants was 10,000 KRW (10 USD). Detailed demographic information of the participants is presented in Table 1.

**Table 1:** Sample demographics.

	**Total sample (*N* = 527)**
	**M (SD)**
Age	38.6 (15.0)
Education (years)	14.6 (3.2)
	*N* (%)
**Gender**
Female	340 (64.5)
Unreported	-
**Marital status**
Single	285 (54.1)
Married	214 (40.6)
Divorced	8 (1.5)
Widowed	10 (1.9)
Unreported	10 (1.9)

### Measures

#### Structured Clinical Interview Instrument (MINI Plus Version 5.0.0)

The MINI is a structured interview instrument for the diagnosis of mental disorders based on the tenth revision of the International Classification of Diseases (World Health Organization, 2004) and the fourth edition of the Diagnostic and Statistical Manual of Mental Disorders (Sheehan et al., 1998). The MINI allows an interviewer to make a diagnostic decision within a 1-h structured interview by following the MINI instructions. The current study used the Korean version of the MINI, which possesses an adequate level of diagnostic accuracy (Yoo et al., 2006). The intra-class correlation coefficient (ICC) of the MINI diagnoses in the current study was 0.92. The MINI was administered by licensed clinical psychologists, psychiatrists, and clinical psychology senior students supervised by licensed clinical psychologists. Interviews generally lasted 30–50 min per participant. Final psychiatric diagnostic decisions were discussed and confirmed by licensed clinical psychologists and a psychiatrist.

#### Beck Anxiety Inventory (BAI)

The BAI is a self-report questionnaire to measure and distinguish anxiety from depressive symptoms (Beck et al., 1988). The BAI includes 21 questions that are answered using a 4-point Likert scale ranging from 0 (not at all) to 3 (severely). In the current study, we adopted a Korean version of the BAI, which has been recently translated by Lee et al. (2016) and is distributed by Pearson Assessments. The validity of the Korean version of the BAI was examined by Oh et al. (2018).

#### Generalized Anxiety Disorder 7-Item Scale (GAD-7), Korean Version

The GAD-7 is a 7-item self-administered instrument to screen for GAD and to assess the severity of symptoms (Spitzer et al., 2006). All respondents were asked to rate their responses on a 4-point Likert scale regarding how frequently they had been disturbed by each presented symptom during the past 2 weeks. The Korean version of the GAD-7 was adopted in the present study. Previous studies have reported excellent reliability (Seo and Park, 2015) and validity (Ahn et al., 2019) of this Korean version.

#### Penn State Worry Questionnaire (PSWQ)

The PSWQ (Meyer et al., 1990) is a 16-item self-administered instrument to measure the frequency and intensity of pathological worry. Each item of the PSWQ is answered using a 5-point Likert scale. In the present study, the Korean version of the PSWQ—translated and examined by Lim et al. (2008)—was adopted, and it possesses good internal consistency (α = 0.85).

#### Mental Health Screening Tool for Anxiety Disorders (MHS: A)

The MHS: A is a 11-item self-report test used to screen GAD and was developed by the authors of this article. Each item of the scale is assessed using a 5-point Likert scale from 0 (not at all) to 4 (always) regarding how respondents experienced each presented symptom during the past 2 weeks. Each item of the MHS: A reflects all the diagnostic criteria of GAD from the DSM-5, with “irritability” being measured with two items.

### Statistical Analysis

The IBM SPSS Statistics 25 statistical program was utilized to calculate the descriptive statistics and perform a correlational analysis and receiver operating characteristic (ROC) curve analysis. Factor analyses and an IRT analysis were performed using the statistical program R (version 3.5.0). The “lavaan” package (Rosseel et al., 2017) was utilized to perform an exploratory factor analysis and a confirmatory factor analysis. Estimation was conducted using the maximum likelihood method. Incremental fit indices and absolute fit indices were utilized to evaluate model fit. Incremental fit indices included the Tucker–Lewis index (TLI) and the comparative fit index (CFI). For absolute model fit, the root mean square error of approximation (RMSEA) and the standardized root mean squared residual (SRMR) were included. Interpretation of model fit indices followed standard criteria (CFI and TLI > 0.90 and RMSEA and SRMR <0.08; Kline, 2005; Hooper et al., 2008). An IRT analysis was performed using the “mirt” package (Chalmers, 2012). A graded response model (GRM) was utilized for the analysis. A GRM is one of the IRT models appropriate for ordered polytomous categories such as Likert scales (Samejima, 1997).

## Results

### Prevalence of General Anxiety Disorder

The average total MHS: A score for all the participants was 9.48 (SD = 10.42) for the offline version and 9.94 (SD = 10.28) for the online version. Among all participants, 50 (9.5% of the sample) were diagnosed as having GAD via the MINI psychiatric structured interview. The means and standard deviations for each item and total scores are presented in Table 2. Among the 50 participants who were diagnosed with GAD, only four were diagnosed as having GAD without comorbid conditions. With regard to comorbidities, 28 participants were diagnosed with major depressive disorder, seven with bipolar disorder, nine with other types of anxiety disorder (e.g., panic disorder), and two with alcohol use disorder. Among all participants, 302 (57.3%) were not diagnosed with any past or current disorder; the remaining 175 were diagnosed with at least one psychiatric condition other than GAD.

**Table 2:** Means, standard deviations, and item–total correlations of the MHS: A.

**Item**	**GAD (*N* = 50) M (SD)**		**Control (*N* = 477) M (SD)**		**Total (*N* = 527) M (SD)**		***r_tot_***		**Ordinal α if item is deleted**	
	**Offline**	**Online**	**Offline**	**Online**	**Offline**	**Online**	**Offline**	**Online**	**Offline**	**Online**
1 Excessive anxiety	2.52 (1.05)	2.56 (1.13)	0.52 (0.81)	0.57 (0.89)	0.71 (1.02)	0.76 (1.08)	0.862^***^	0.845^***^	0.97	0.97
2 Uncontrollable worry	2.58 (1.05)	2.82 (0.98)	0.70 (0.98)	0.75 (0.98)	0.88 (1.13)	0.95 (1.15)	0.847^***^	0.865^***^	0.97	0.97
3 Restlessness	2.52 (1.18)	2.50 (1.06)	0.51 (0.83)	0.60 (0.88)	0.70 (1.05)	0.78 (1.05)	0.872^***^	0.868^***^	0.97	0.97
4 Fatigue	2.67 (1.16)	2.46 (1.11)	0.76 (1.03)	0.77 (0.97)	0.94 (1.18)	0.93 (1.10)	0.845^***^	0.799^***^	0.97	0.97
5 Difficulty paying attention	2.48 (1.13)	2.52 (1.00)	0.57 (0.89)	0.62 (0.88)	0.75 (1.08)	0.80 (1.05)	0.833^***^	0.838^***^	0.97	0.97
6 Irritability	2.58 (1.18)	2.56 (1.05)	0.67 (1.01)	0.74 (0.99)	0.85 (1.17)	0.91 (1.13)	0.857^***^	0.854^***^	0.97	0.97
7 Muscle tension	2.58 (1.25)	2.62 (1.18)	1.01 (1.16)	1.05 (1.12)	1.16 (1.26)	1.20 (1.21)	0.790^***^	0.801^***^	0.97	0.97
8 Insomnia	2.90 (1.23)	2.74 (1.19)	1.01 (1.18)	1.06 (1.16)	1.19 (1.31)	1.22 (1.26)	0.785^***^	0.767^***^	0.97	0.97
9 Impairment in daily function	2.46 (1.15)	2.58 (1.16)	0.36 (0.77)	0.45 (0.88)	0.56 (1.02)	0.65 (1.11)	0.843^***^	0.857^***^	0.97	0.97
10 Chest discomfort	2.42 (1.21)	2.46 (0.99)	0.68 (0.99)	0.61 (0.92)	0.85 (1.14)	0.78 (1.08)	0.817^***^	0.821^***^	0.97	0.97
11 Feeling on edge	2.64 (1.12)	2.74 (0.83)	0.71 (0.96)	0.77 (0.96)	0.89 (1.13)	0.96 (1.11)	0.862^***^	0.870^***^	0.97	0.97
MHS: A Total	28.30 (9.96)	28.56 (8.44)	7.50 (8.28)	7.99 (8.32)	9.48 (10.42)	9.94 (10.28)	0.908^***^^a^		-	-

*** *P <0.001, GAD, generalized anxiety disorder; a, correlational coefficient between the online version and offline version of the MHS: A*.

### Internal Consistency and Convergent Validity

To identify the internal reliability of the MHS: A, the ordinal alpha was calculated based on the polychoric correlation matrix. The analysis procedure suggested by Gadermann et al. (2012) was applied, and the R package “psych” was utilized for the analysis (Revelle and Revelle, 2015). Both offline and online versions of the MHS: A had an ordinal alpha of 0.97, indicating a high level of internal reliability. Furthermore, the coefficients of the ordinal alpha remained the same even if individual items were deleted from both the offline and online versions of the scale, suggesting that there was no significant benefit from excluding any individual items (Table 2). The means, standard deviations, and item–total correlations for the offline- and online-based MHS: A are presented in Table 2. Item–total correlations ranged from 0.767 to 0.872, indicating good internal consistency. A correlational analysis with each item was conducted, and the correlation ranged from 0.533 to 0.822. Details on the correlational coefficients are presented in Supplementary Tables 1, 2. The online and offline versions of the MHS: A had a correlational coefficient of 0.908, proving that the two scales were virtually identical.

To examine convergent validity, correlational analyses with other anxiety scales were conducted. The MHS: A total score was significantly correlated with the BAI total score (*r* = 0.832 with the online version, *r* =0.827 with the offline version, *p* < 0.001), GAD-7 total score (*r* = 0.828 with the online version, *r* = 0.870 with the offline version, *p* < 0.001), and PSWQ total score (*r* = 0.666 with the online version, *r* = 0.700 with the offline version, *p* < 0.001), indicating good convergent validity.

### Factor Structure

**Figure 2:**
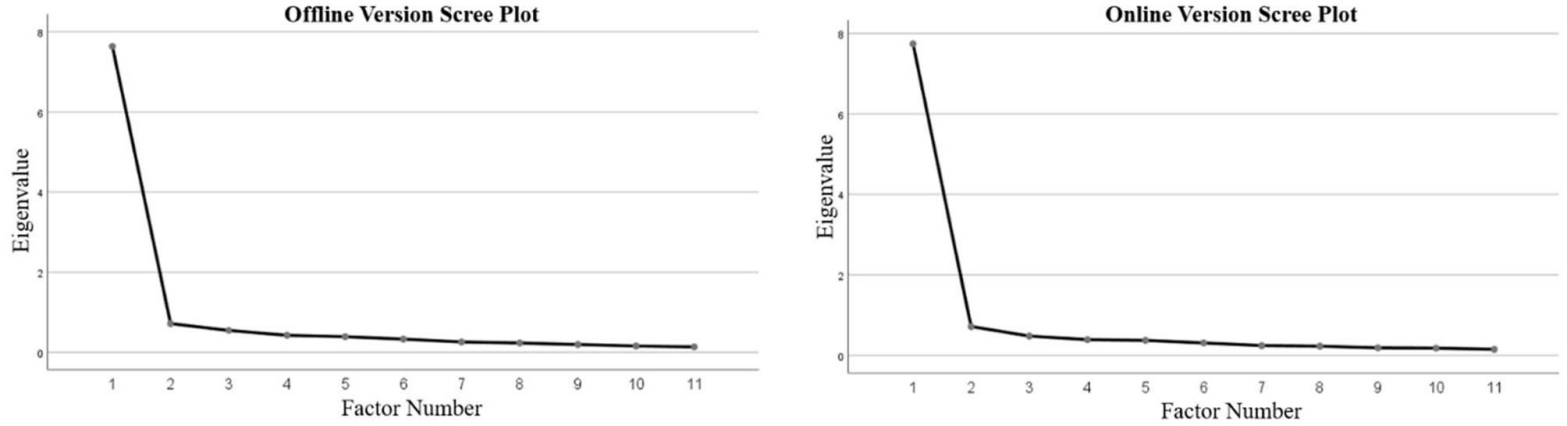
Scree plots of the MHS: A.

To test the factor structure of the MHS: A, both exploratory and confirmatory factor analyses were performed. Data were randomly assigned to two groups. An exploratory factor analysis (EFA) was performed with half the data. The principal axis factoring method was applied for the EFA. The result of the analysis suggested a one-factor model for both the online and offline versions of the MHS: A. The total explained variance is presented in Table 3, and the Scree plots are presented in Figure 2.

**Table 3:** Total explained variance for the offline and online versions of the MHS: A.

**Factor**	**Offline Version**		**Online Version**	
	**Initial Eigenvalues Total**	**Initial Eigenvalues Percent of Variance**	**Initial Eigenvalues Total**	**Initial Eigenvalues Percent of Variance**
1	7.639	69.443	7.738	70.346
2	0.714	6.492	0.717	6.515
3	0.544	4.945	0.480	4.364
4	0.422	3.839	0.391	3.556
5	0.388	3.525	0.373	3.389
6	0.326	2.966	0.310	2.815
7	0.257	2.334	0.244	2.215
8	0.230	2.091	0.229	2.085
9	0.192	1.747	0.189	1.716
10	0.155	1.409	0.180	1.639
11	0.133	1.209	0.150	1.362

*Extraction method: principal axis functioning*.

The factor loadings for individual items for both the offline and online versions are summarized in Supplementary Table 3.

**Figure 3:**
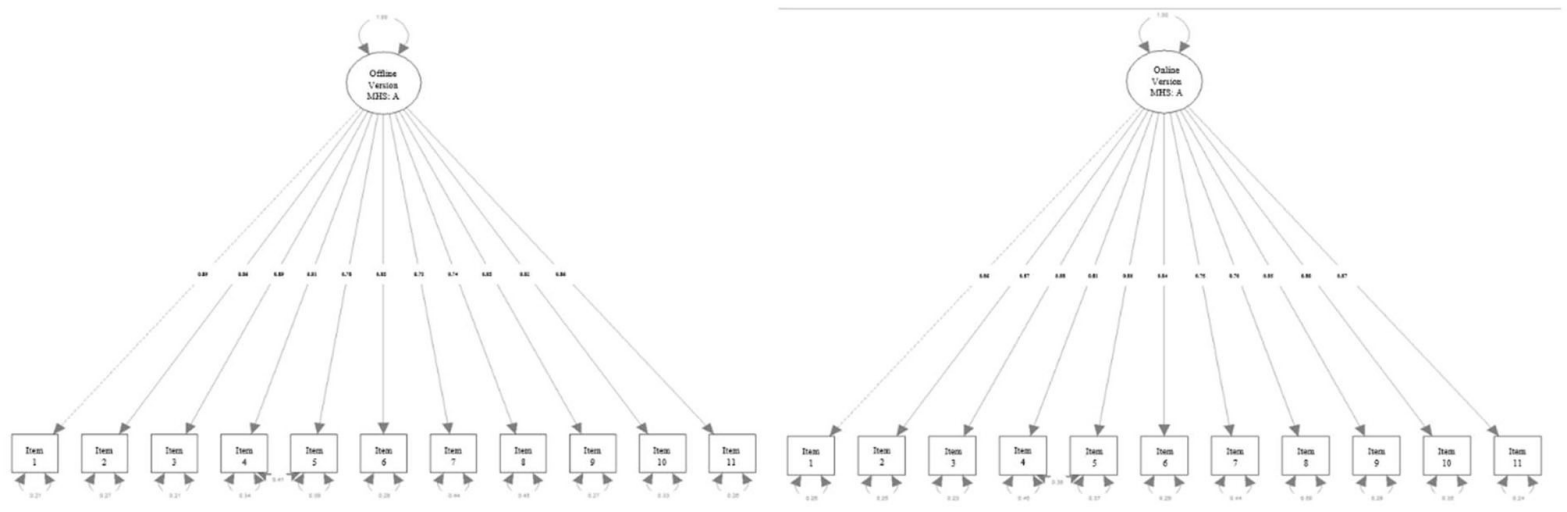
Factor structure of the MHS: A.

A confirmatory factor analysis was performed with the remaining data. The exploratory structural equation modeling (ESEM) method was also applied to the traditional CFA method, as recommended by Marsh et al. (2009). An inspection of the modification indices (MIs) suggested correlating the residuals of Items 4 and 5 could improve the model fit for both the offline and online versions of the MHS: A, and this suggestion was adopted. Details on the CFA fit indices are presented in Table 4, and the factor models are depicted in Figure 3. The result of the one-factor factor analysis showed reasonable model fit indices for both the online and offline versions of the scale. Both the TLI and CFI met the criteria. Although the criterion for the RMSEA was not satisfied, the criterion for the SRMR was satisfied for both the offline and online versions. Information indices were not interpreted since there was no other model to which this model could be compared.

**Table 4:** Summary of Goodness-of-Fit Indices for CFA.

	**Fit indices**								
**Model tested**	***χ^2^***	**AIC**	**BIC**	**aBIC**	**CFI**	**TLI**	**SRMR**	**RMSEA**	**90% CI**
Offline Version 1-Factor Model	197.313^***^ (*df* = 43)	6323.995	6406.067	6333.147	0.944	0.929	0.038	0.117	0.101–0.134
Online Version 1-Factor Model	312.713^***^ (*df* = 43)	12842.273	12940.419	12867.411	0.948	0.934	0.038	0.109	0.098–0.121

*AIC, Akaike information criterion; BIC, Bayesian information criterion; aBIC, sample-size adjusted BIC; CFI, comparative fit index; TLI, Tucker-Lewis index; SRMR, standardized root mean squared residual; RMSEA, root mean square error of approximation; CI, confidence interval*.

*** *P < 0.001*.

### Criterion Validity

**Figure 4:**
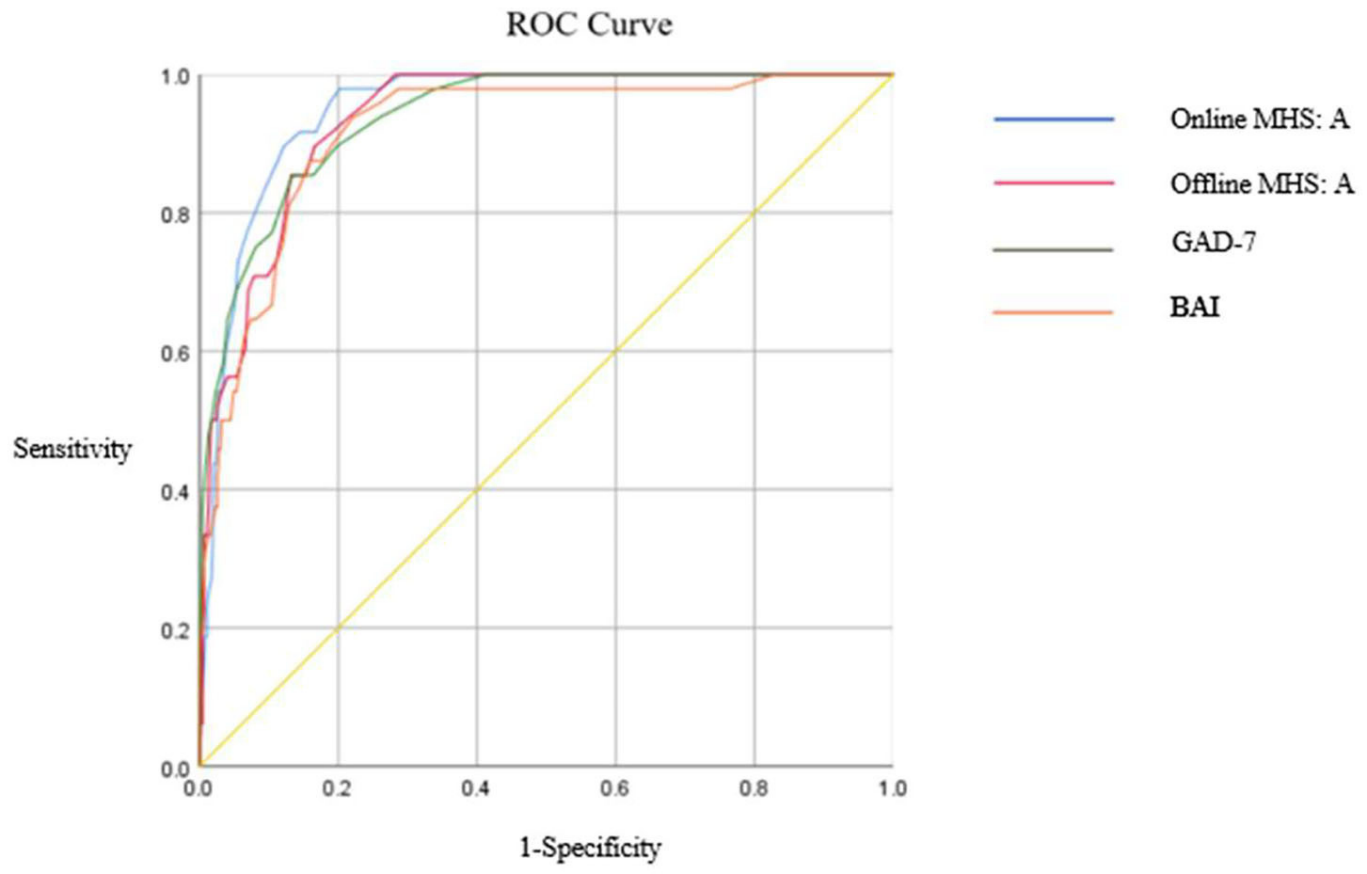
ROC curve for three different anxiety measures.

ROC analyses were conducted to examine the criterion validity of the online and offline versions of the MHS: A. To compare screening capabilities, an ROC analysis was also conducted with the BAI and GAD-7. The ROC curves for the four measures are depicted in Figure 4, and detailed results are presented in Table 5. Both online and offline versions of the MHS: A showed a greater area under the curve (AUC) for detecting GAD than the BAI and GAD-7. Youden's index (Youden's index J = sensitivity + specificity – 1; Youden, 1950) was utilized to calculate the optimal cut-off points for detecting GAD, and a score of 15 was identified as the optimal cut-off score for both the online and offline versions of the MHS: A to detect GAD.

**Table 5:** Results of ROC analyses for GAD.

**Measures and cut-off score**	**AUC**	**SEN**	**SPE**	**PPV**	**NPV**
Offline MHS: A	0.938	0.900	0.834	0.363	0.988
Online MHS: A	0.950	0.980	0.800	0.338	0.997
BAI Mild =10	0.919	0.958	0.739	0.272	0.994
BAI Moderate = 19		0.667	896	0.395	0.964
BAI Severe = 30		0.458	0.300	0.611	0.946
GAD-7 Mild = 5	0.938	0.979	0.659	0.232	0.997
GAD-7 Moderate = 10		0.771	0.894	0.438	0.975
GAD-7 severe = 15		0.542	0.975	0.684	0.951

*AUC, area under the curve; CI, confidence interval; MDD, major depressive disorder; DD, depressive related disorder; SEN, sensitivity; SPE, specificity; J, Youden's index; PPV, positive predictive value; NPV, negative predictive value*.

This optimal cut-off score for the online version of the MHS: A showed a 0.980 sensitivity and 0.800 specificity, and for the offline version showed a 0.900 sensitivity and 0.834 specificity. Compared to the BAI and GAD-7's mild, moderate, and severe cut-off points, both the offline and online versions of the MHS: A performed better in screening GAD.

To verify the GAD discrimination ability of each item, an ROC analysis was performed separately. The AUC for each item ranged from 0.82 to 0.92. The item with the highest AUC value was “feeling on edge” for the online version, and “impairment in daily function” for the offline version, while the item with the lowest AUC was “muscle tension” for both versions. Details on the AUC values for each item are presented in Supplementary Table 4.

### Item Response Theory Analyses

**Figure 5:**
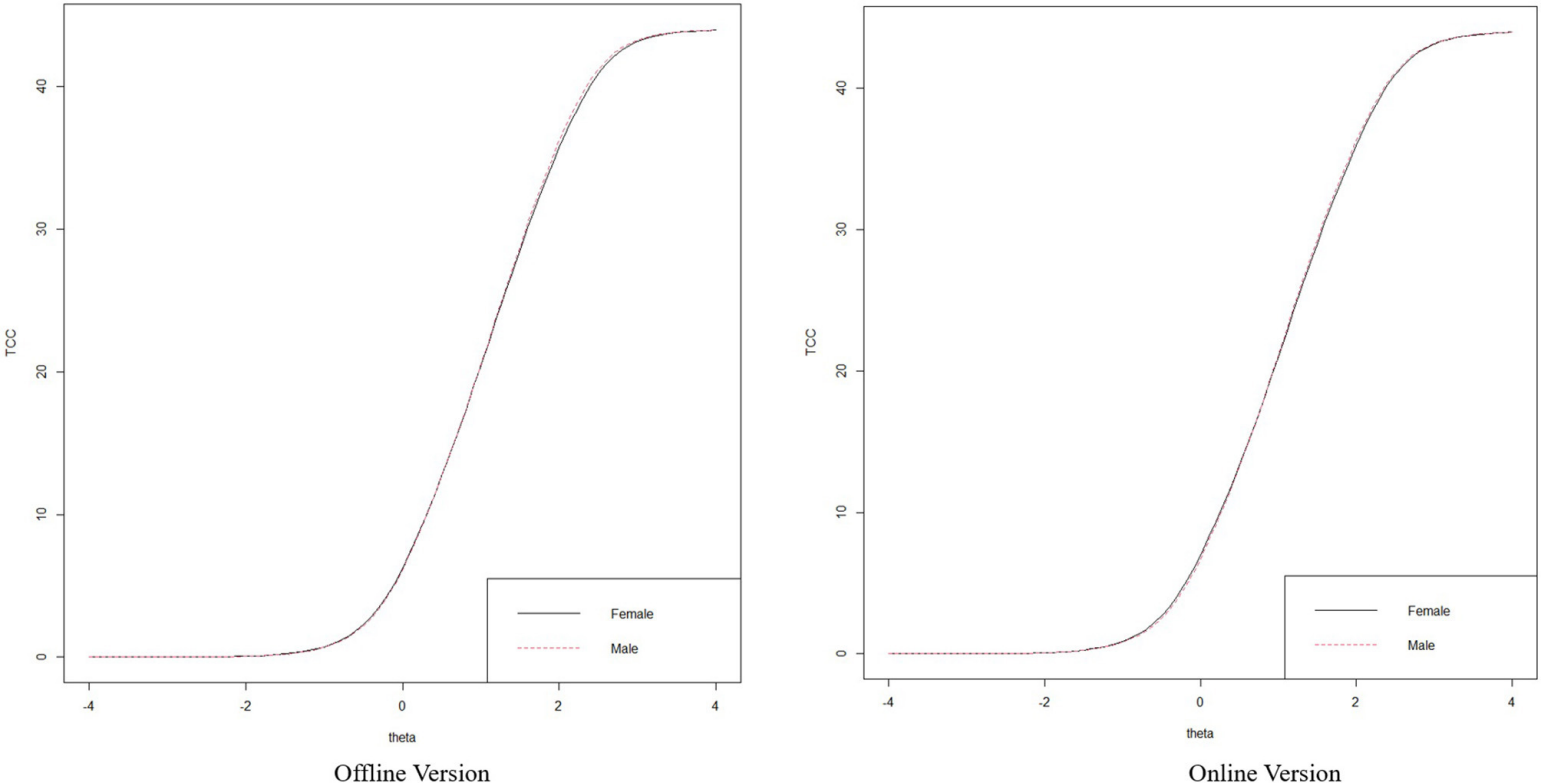
TCC based on gender difference.

A polytomous IRT analysis was conducted to evaluate each item's suitability. Each item's parameters are presented in Table 6, and the item characteristic curves for each item are depicted in Supplementary Figures 1, 2. As mentioned previously, the weights for each item were calculated by averaging the difficulty parameters of each item, and these are also presented in Table 5. Item discriminability ranged from 2.21 to 4.23, indicating very good discriminatory power. For the difficulty parameters, the question boundary parameters in each item showed an appropriate amount of spacing, without overlapping or transposition. The obtained test information curves (TICs) are depicted in Supplementary Figure 3. The TIC of the MHS: A formed a peak-like line at the area around 0–2.0 standard deviations. After the 2.0 standard deviation point, information decreased sharply, indicating that the MHS: A is more suitable as a screening tool rather than as a measure of severity. A differential functioning (DIF) analysis was performed to compare item functioning between genders. The DIF analysis was conducted using the lordif package with the statistical program R (Choi et al., 2011). We used the likelihood ratio (LR) χ^2^ test as the detection criterion at the α level of 0.01. The analysis suggested two items for the offline version of the scale—item 6: Pr(χ^2^_12_, 1) = 0.0055, *R*^2^_12_ = 0.0059, (β_1_) = 0.0167, Pr(χ^2^_13_, 2) = 0.0155, *R*^2^_13_ = 0.0064, Pr(χ^2^_23_, 1) = 0.4349, *R*^2^_23_ = 0.0005 and item 9: Pr(χ^2^_12_, 1) = 0.0021, *R*^2^_12_ = 0.0091, (β_1_) = 0.0127, Pr(χ^2^_13_, 2) = 0.0081, *R*^2^_13_ = 0.0093, Pr(χ^2^_23_, 1) = 0.6771, *R*^2^_23_ = 0.0002—and one item for the online version—item 6: Pr(χ^2^_12_, 1) = 0.0408, *R*^2^_12_ = 0.0031, (β_1_) = 0.0031, Pr(χ^2^_13_, 2) = 0.0011, *R*^2^_13_ = 0.01, Pr(χ^2^_23_, 1) = 0.0022, *R*^2^_23_ = 0.0069—displayed gender-related differences. However, the density–weighted impact was negligible for all three items because few subjects had that trait level in the research population. Figure 5 illustrates the test characteristic curves for female and male individuals. These curves suggest that at the overall test level, there is minimal difference in the total expected score at any anxiety level for female and male individuals.

**Table 6:** Item parameters of each item.

	**a**		**b1**		**b2**		**b3**		**b4**		**Weight**	
	**Offline Version**	**Online Version**	**Offline Version**	**Online Version**	**Offline Version**	**Online Version**	**Offline Version**	**Online Version**	**Offline Version**	**Online Version**	**Offline Version**	**Online Version**
Item1	4.00	3.64	0.14	0.17	0.97	0.89	1.50	1.37	2.34	2.19	1.24	1.15
Item2	3.36	3.77	0.01	−0.07	0.73	0.66	1.39	1.24	2.13	1.98	1.06	0.95
Item3	4.18	4.23	0.24	0.08	0.88	0.89	1.53	1.43	2.16	2.13	1.20	1.13
Item4	2.74	2.49	−0.06	−0.12	0.73	0.75	1.30	1.46	1.98	2.35	0.99	1.11
Item5	2.84	2.95	0.19	0.05	0.96	0.92	1.55	1.54	2.32	2.33	1.26	1.21
Item6	3.31	3.45	0.10	−0.07	0.80	0.73	1.27	1.32	1.97	1.98	1.04	0.99
Item7	2.22	2.34	−0.30	−0.43	0.53	0.49	1.07	1.11	2.04	2.00	0.83	0.79
Item8	2.22	2.21	−0.27	−0.40	0.51	0.49	1.07	1.14	1.78	1.85	0.77	0.77
Item9	3.72	3.97	0.49	0.42	1.15	0.99	1.58	1.41	2.15	2.00	1.34	1.20
Item10	2.71	2.91	0.09	0.15	0.78	0.84	1.45	1.49	2.24	2.44	1.14	1.23
Item11	3.65	3.83	−0.03	−0.17	0.74	0.70	1.40	1.29	1.96	2.03	1.02	0.96

*a, discriminability value; b1–b4, boundary parameter*.

## Discussion

The present study was the third phase of a mental health screening tool development project, in which we examined the psychometric properties and diagnostic screening utility of the MHS: A with 527 Korean community samples. Overall, the MHS: A is a psychometrically sound GAD screening measure. It demonstrated excellent internal consistency and good convergent validity with other anxiety measures such as the GAD-7, BAI, and PSWQ. The EFA and CFA results confirmed that the MHS: A had a one-factor structure. The criteria for the TLI, CFI and SRMR were satisfied for both the online and offline versions of the scale, but the RMSEA did not meet the criterion. However, disagreements between the RMSEA and CFI may occur, and since such discrepancy is not diagnostic of specific problems with the model specifications or data (Lai and Green, 2016), this one-factor model can be acceptable when considering other fit indices.

The MHS: A revealed excellent diagnostic accuracy for GAD detection. There was an optimal balance between the sensitivity and specificity of the MHS: A when the total cut-off score was set at 15.7 or above, indicating better performance than the GAD-7 and BAI. Furthermore, the diagnostic accuracy of the MHS: A based on an ROC analysis was better than that of the GAD-7 and the BAI for GAD screening.

The MHS: A encompasses all the DSM-5 diagnostic criteria for GAD, unlike other tools for anxiety disorders (i.e., GAD-7, BAI, PWSQ), as the MHS: A includes not only the cognitive and physical symptoms of GAD but also the “impairment of functioning” domain. As Titov et al. (2011) indicated, a test covering all diagnostic criteria is more useful in identifying remission, improvement, and recovery of mental disorders. Therefore, the MHS: A would be more suitable as a diagnostic screening tool over the course of prevention and treatment.

The results of the IRT analysis showed that item discriminability was very good, indicating that each item had its own informative value and offers high information values across different anxiety levels. The TIC, which provides information on how an instrument would work in estimating person locations, had a peak-like shape between 0.0 and 2.0 standard deviations, with the highest point occurring at around 1.5 standard deviations and a sharp decrease after 2.0 standard deviations above the mean. In other words, the MHS: A can provide maximum information about diagnostic decisions with the highest reliability and the lowest standard error, ranging from anxiety severity of the average population to the top 2–3 percentile of the population. In particular, it best predicts levels of anxiety of people belonging to the top 7 percentile, which is consistent with the GAD group we aimed to screen. The GAD lifetime prevalence rate in Korea is reported to be 2.4% (Hong et al., 2017), and the MHS: A, which measures from the average to diagnosable level of anxiety, is considered to have adequate psychometric properties to be used as a screening tool. Given that the GAD-7 should be reconsidered as a screening test due to its difficulty in discriminating the lower spectrum of anxiety (Jordan et al., 2017), the MHS: A could serve as an alternative screening tool, as it is constructed with the best combination of items that provide optimal information, based on the discrimination value of each item.

Regarding the AUC analysis for each item, item 11 (“I was nervous or tense.”) showed the highest AUC value, followed by item 2 (“I could not control or stop worrying.”). This result is consistent with a previous study that found a remarkable number of Korean patients with GAD who complained of symptoms related to autonomic nervous system imbalances, such as insomnia, and reduced adaptability in the body toward environmental changes (Choo et al., 2005). In addition, these two items are similar to the GAD-2, which comprises core anxiety items from the GAD-7, and are considered to reflect the clinical features of GAD. In addition, “chest oppressed,” which did not appear in previous assessments for anxiety disorders, is one of the key physical features in Hwa-byung (i.e., a Korean culture-specific psychiatric condition). Given that cultural differences can affect the administration and interpretation of an assessment (Parkerson et al., 2015), the MHS: A could capture the anxiety symptoms of Koreans.

Some limitations and implications for future studies should be noted. First, although we included an item related to social and occupational dysfunction in the MHS: A, no additional measures to assess functional impairments were included in this study. The TIC analysis showed that the MHS: A had better psychometric properties as a screening tool than other anxiety measures; thus, a future study should investigate whether the MHS: A would also reflect the functioning impairment level of community-dwelling individuals with GAD. Second, we did not measure test-retest reliability. Future studies should report the stability of scores over time. Finally, since our samples were limited to Koreans, further research is needed to investigate the generalizability of the current findings with other samples.

Despite these limitations, the MHS: A can be used as an acceptable and clinically efficient screening tool for GAD in Korea. It is designed with a focus on the characteristic symptoms and item response patterns of Koreans, providing proper clinical information with a small number of items. The excellent diagnostic accuracy of the MHS: A could also help relieve the substantial economic and psychological impact on patients as well as the burden on community healthcare systems. Given the low rate of detection of GAD among non-psychological experts (i.e., family physicians) and the considerably large amount of time that elapses before patients receive effective treatment (Wagner et al., 2006), the diagnostic accuracy of the MHS: A could help in decision-making that would prevent delays in proper therapeutic interventions due to diagnostic errors and enhance the effectiveness of treatment through early intervention (Altamura et al., 2008; Bereza et al., 2009). In addition, the MHS: A is available on both online and offline platforms, and it is also advantageous in that it can be flexibly administered according to the environment in which the test is conducted or the screening test method preferred by participants. Recently, due to COVID-19, the importance and clinical utility of telehealth psychiatric evaluation has increased. Hence, the MHS: A could be considered an efficient screening tool for diagnostic decision-making for GAD in non-face-to-face situations.

## Data Availability Statement

The raw data supporting the conclusions of this article will be made available by the authors, without undue reservation.

## Ethics Statement

The studies involving human participants were reviewed and approved by Institutional Review Boards of the Korea University [1040548-KU-IRB-15-92-A-1(R-A-1)(R-A-2)(R-A-2)] and the Ilsan Paik Hospital [ISPAIK 2015-05-221-009]. The patients/participants provided their written informed consent to participate in this study.

## Author Contributions

S-HK, KP, YC, S-HL, and K-HC devised the study, main conceptual ideas, and the study process. K-HC supervised the overall study process and direction. S-HK, KP, and SY contributed to the data collection, methodology, and the writing of the manuscript. K-HC reviewed and supervised the drafting of the manuscript. All authors contributed to and approved the final version of the manuscript.

## Conflict of Interest

The authors declare that the research was conducted in the absence of any commercial or financial relationships that could be construed as a potential conflict of interest.
