# Supplementary material for: A Brief Online and Offline (Paper-and-Pencil) Screening Tool for Generalized Anxiety Disorder: The Final Phase in the Development and Validation of the Mental Health Screening Tool for Anxiety Disorders (MHS: A)
Source: Front Psychol. 2021 Feb 22;12:639366. doi: 10.3389/fpsyg.2021.639366 (PMC7937919; doi:10.3389/fpsyg.2021.639366)
Supplement: Supplementary file 1 [file Table_1.pdf]

Supplementary Table 1. Correlation between each item – Offline version

	1	2	3	4	5	6	7	8	9	10
1 Excessive anxiety	1									
2 Uncontrollable worry	.801**	1								
3 Restlessness	.822**	.806**	1							
4 Fatigue	.667**	.657**	.690**	1						
5 Attention difficulty	.688**	.647**	.694**	.781**	1					
6 Irritability	.711**	.702**	.706**	.695**	.710**	1				
7 Muscle tension	.572**	.620**	.586**	.669**	.595**	.672**	1			
8 Insomnia	.568**	.585**	.596**	.639**	.605**	.618**	.680**	1		
9 Impairment in daily function	.769**	.674**	.754**	.669**	.718**	.713**	.555**	.600**	1	
10 Chest discomfort	.691**	.656**	.671**	.647**	.616**	.623**	.610**	.618**	.657**	1
11 Feeling on edge	.732**	.688**	.757**	.660**	.639**	.748**	.635**	.634**	.722**	.735**

Supplementary Table 2. Correlation between each item – Online version

	1	2	3	4	5	6	7	8	9	10
1 Excessive anxiety	1									
2 Uncontrollable worry	.788***	1								
3 Restlessness	.801***	.787***	1							
4 Fatigue	.579***	.619***	.631***	1						
5 Attention difficulty	.639***	.680***	.681***	.743***	1					
6 Irritability	.717***	.710***	.716***	.623***	.689***	1				
7 Muscle tension	.564***	.619***	.601***	.667***	.616***	.692***	1			
8 Insomnia	.533***	.595***	.569***	.620***	.645***	.587***	.654***	1		
9 Impairment in daily function	.741***	.736***	.744***	.646***	.729***	.702***	.602***	.600***	1	
10 Chest discomfort	.685***	.692***	.686***	.602***	.649***	.649***	.637***	.555***	.682***	1
11 Feeling on edge	.748***	.727***	.786***	.613***	.650***	.762***	.659***	.620***	.712***	.728***

Supplementary Table 3. Factor loading from EFA

	Offline version<br>Factor Loading	Online version<br>Factor Loading
1 Excessive anxiety	0.865	0.835
2 Uncontrollable worry	0.835	0.858
3 Restlessness	0.876	0.874
4 Fatigue	0.808	0.755
5 Attention difficulty	0.825	0.807
6 Irritability	0.842	0.832
7 Muscle tension	0.734	0.743
8 Insomnia	0.724	0.769
9 Impairment in daily function	0.829	0.861
10 Chest discomfort	0.764	0.81
11 Feeling on edge	0.851	0.876

Supplementary Table 4. AUC for each item

	MHS: A Offline AUC	MHS: A Online AUC
1 Excessive anxiety	0.915	0.899
2 Uncontrollable worry	0.888	0.911
3 Restlessness	0.911	0.903
4 Fatigue	0.874	0.859
5 Attention difficulty	0.889	0.903
6 Irritability	0.879	0.885
7 Muscle tension	0.820	0.820
8 Insomnia	0.852	0.829
9 Impairment in daily function	0.922	0.905
10 Chest discomfort	0.855	0.897
11 Feeling on edge	0.896	0.920

Supplementary Figure 1. ICC for each item of Offline version MHS: A

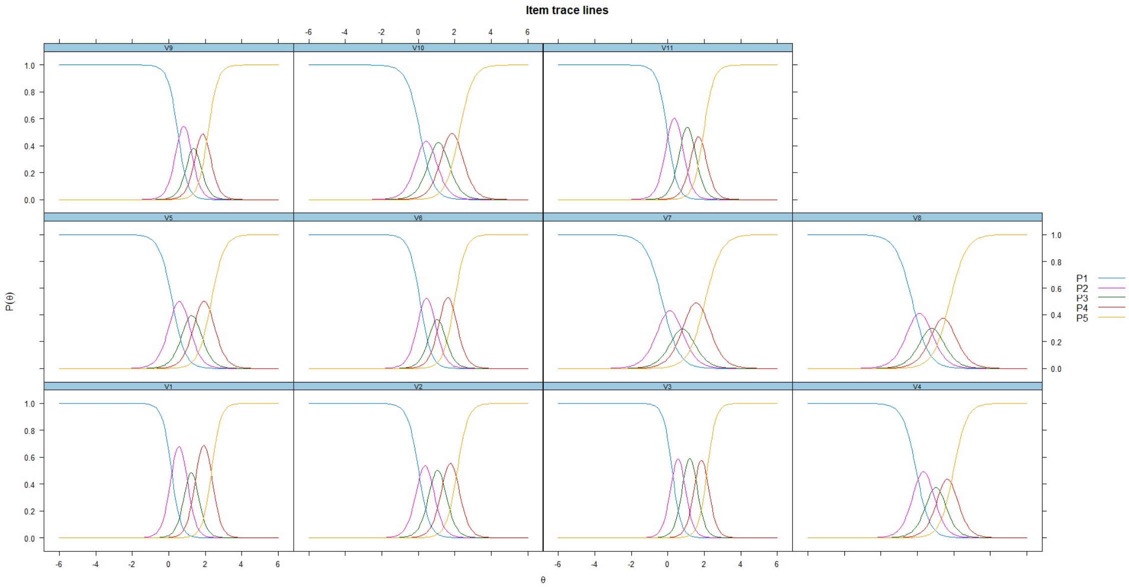

Note. P(θ): probability of a response in the given category across different anxiety levels, θ: level of anxiety expressed in terms of standard deviation

Supplementary Figure 2. ICC for each item of Online version MHS: A

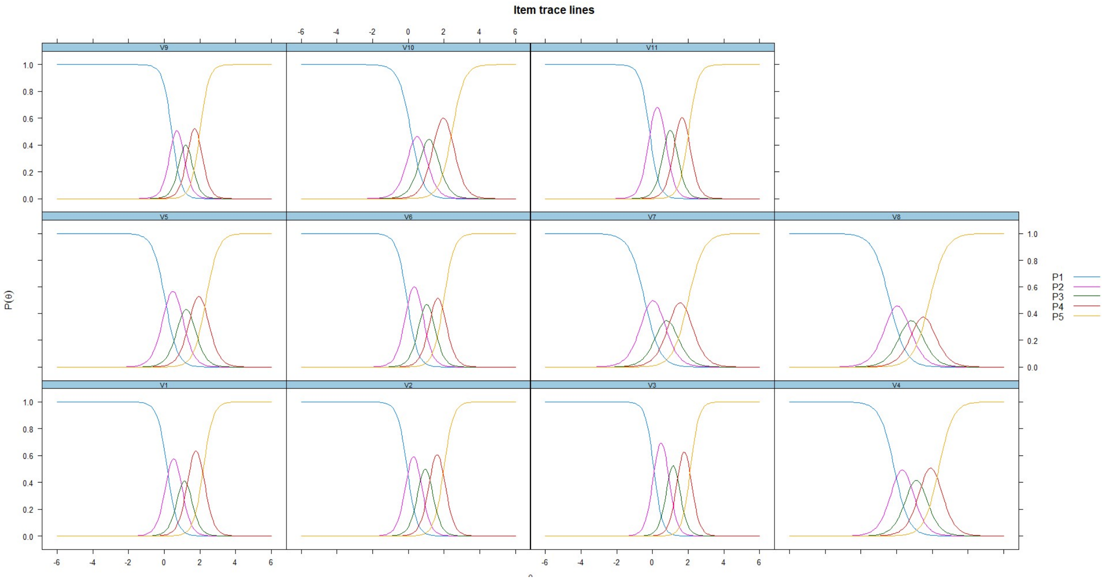

Note. P(θ): probability of a response in the given category across different anxiety levels, θ: level of anxiety expressed in terms of standard deviation

Supplementary Figure 3. TIC for Offline and Online version of MHS: A

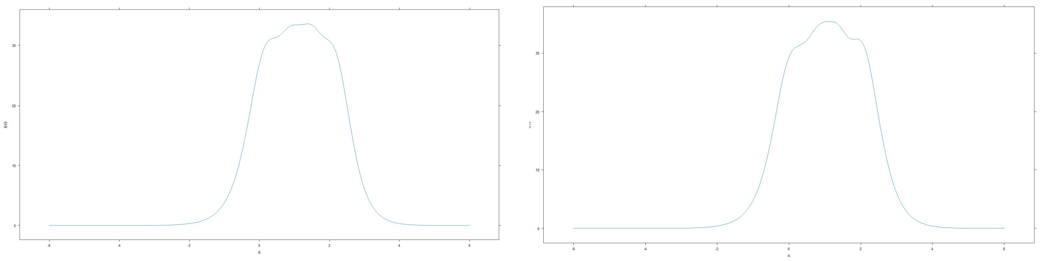

Test Information Curve for Offline MHS: A

Test Information Curve for Online MHS: A

Note. I(θ): Information value, θ: level of anxiety expressed in terms of standard deviation
